# Historical Highlight: Three Seminal Articles Relating to the Recognition of Protein Antigens by Antibodies

**DOI:** 10.20411/pai.v11i2.1091

**Published:** 2026-09-30

**Authors:** Neil S. Greenspan, Emily N. Kukan

**Affiliations:** 1 Case Western Reserve University

**Keywords:** Complementarity, Hypervariable Regions, Complementarity Determining Regions, Epitope, Paratope

“… I agree that there is a problem of evaluating thermodynamic quantities, such as standard free energy changes, from the analysis of a crystallographic structure for the complex of receptor and ligand. The information provided by a crystal structure may, however, be of significant value.”

Personal communication from Linus Pauling to NSG (March 25,1994).

## INTRODUCTION

Beginning with this installment, the *Pathogens and Immunity* Historical Highlight series will focus, at least in some instances, on important themes in immunology, infectious disease, and microbiology by summarizing the contributions of a small and highly selected sampling of foundational articles instead of discussing just one landmark publication. By contrast, the first 2 installments were devoted to single studies that offered insights fundamental to all 3 of the above-cited disciplines and many other biomedical fields.

This commentary will address three papers pertaining to antigen-specific immunological recognition that offer notable insights. Since antigen specificity was first described and extensively explored for antibodies, the focus will be on the factors that influence the noncovalent binding to and discrimination among potential ligands exhibited by these key glycoprotein mediators of humoral immunity.

Of course, many other sets of 3 articles could have been selected with the same purpose in mind. Nevertheless, we hope our discussion of the 3 articles we chose will be informative and perhaps offer a constellation of insights that extend beyond what is offered in immunology textbooks or the vast majority of articles in the immunological literature.

## PAULING AND DELBRÜCK

We start in 1940 when Linus Pauling and Max Delbrück published a short commentary including no new data in *Science* [1]. Pauling was recognized as the leading exponent of quantum chemistry in the United States and an expert in using biophysical methods to determine the structures of chemical compounds. Max Delbrück was a physicist by background. After beginning his graduate training in astronomy and astrophysics, Delbrück switched his focus to theoretical physics, especially quantum physics, under the guidance of Wolfgang Pauli and then Niels Bohr at Bohr’s institute in Copenhagen.

After becoming dissatisfied with his progress as a physicist, Delbrück decided to transition into biology [2]. In particular, he chose to focus on aspects of bacteriophage biology in part because he became interested in pursuing Bohr’s speculation about applying the complementarity principle to living systems [3]. In this context, Delbrück noted that bacterial viruses were among the simplest entities that displayed some of the salient features of living systems, such as replication.

The joint article by Pauling and Delbrück was specifically motivated by the desire to criticize the proposal by the German quantum physicist Pascual Jordan that in biology, molecular interactions would primarily involve identical structures. In dramatic contrast, Pauling and Delbrück proposed that interactions between biomolecules would occur between structures that exhibited shape “complementariness” (which is now typically called “complementarity”) for one another.

In the last decades of the nineteenth century and the first decade of the twentieth century, Emil Fischer (working on enzymes), J.N. Langley (focused on cellular host molecules that interacted with physiologically consequential drugs), and Paul Ehrlich (seeking to understand serum anti-toxins) developed concepts that eventually gave rise to Pauling’s concept of complementariness [4]. Pauling and Delbrück may have been the first to update this concept with the perspectives derived from quantum chemistry, including the nature of chemical bonds. Insights into covalent bonds were being discovered in conjunction with the recently acquired abilities to determine molecular structures at atomic resolution using X-ray crystallography and electron diffraction. For the first time, investigators could determine the 3-dimensional distributions of atoms in space for both organic and inorganic molecules of modest size.

Since 1940, the concept of complementarity has been revealed to be a family of concepts including shape complementarity, chemical complementarity, and electrostatic complementarity. Furthermore, the question of how to quantitatively assess the extent of shape complementarity has been shown to have an impressive variety of answers, such as: 1) the area of the 2 surfaces of the interacting proteins that are buried by the formation of the complex, 2) the extent to which water molecules are excluded from the interface, 3) the magnitude of the packing density for the interface atoms, and 4) the degree of shape correlation calculated for every antibody or antigen atom deemed to be in the interface [5]. Other reasonable measures of shape complementarity may well currently exist or be devised in the future.

An even more basic complexity is that there are multiple definitions of a protein’s surface [6] (Figure 1). The *atomic surface* is simply the surfaces of atoms as defined by their respective van der Waals radii [7]. The *molecular surface* smoothly extends over tiny (on the atomic scale) crevices between atoms [8]. The *solvent-accessible surface* corresponds to the line traced out by the center of a sphere the size of a water molecule (radius of about 1.4 Å) rolled over the protein exterior [7]. According to each of these definitions of the protein (or other biological macromolecule) surface, the resulting shape at high resolution differs.

**Figure 1.**
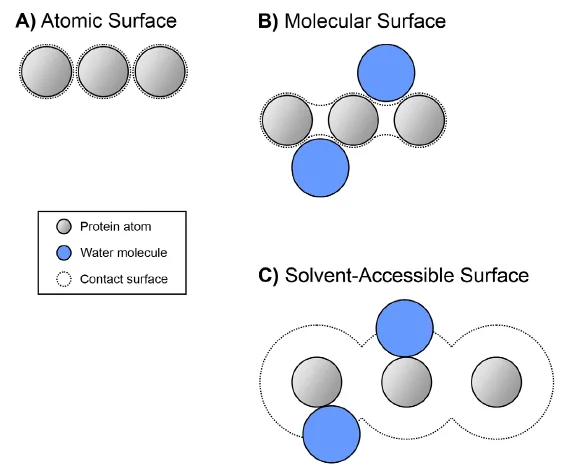
Schematic illustration of protein surfaces according to three different definitions, including (A) atomic, (B) molecular, and (C) solvent-accessible.

Another factor not dealt with in the 1940 article by Pauling and Delbrück is the role of solvent molecules, which are typically water molecules in biological systems. In addition to the molecules of bulk solvent, some water molecules, referred to as “bound” water molecules, are found to persist for relatively long time intervals (“dwell times”) at precise locations on a protein surface or at an interface between a protein and a ligand [9]. Subsequent studies using methods not available in 1940 revealed that bound water molecules and counter ions can substantially influence both the affinity and specificity of biomolecular interactions, including those involving proteins binding either other proteins or small molecule ligands (see below in discussion of the complex between the D1.3 monoclonal antibody and hen egg lysozyme) [10].

## WU AND KABAT

Thirty years after the commentary by Pauling and Delbrück, in 1970, Elvin Kabat and Tai Te Wu published an informative analysis of antibody amino acid sequence variation in the *Journal of Experimental Medicine* [11]. Their study focused on the amino acid sequence variation of the N-terminal halves (what we now term the variable domain) of mouse kappa and human kappa and lambda Bence Jones proteins, the light chains of monoclonal immunoglobulins (also referred to as paraproteins). Bence Jones proteins are found in the urine of some patients (or mice) with plasma cell malignancies such as, in humans, multiple myeloma and Waldenström macroglobulinemia. This paper represents an early instance of a bioinformatic investigation based on data collected from many prior experimental investigations.

Wu and Kabat were the first to define what we now know as hypervariable and framework amino acid residues in the heavy-chain (H) and light-chain (L) variable (V) domains (ie, VH or VL) of antibodies and their analogues in T cell receptors. This classification arises out of the definition of “variability” applied to each position in the amino acid sequences of the human and mouse V domains of the proteins identified in the preceding paragraph. Without any explicit effort to derive the formula for amino acid sequence “variability” at each position in the linear polypeptide sequence, the authors defined this concept as follows:



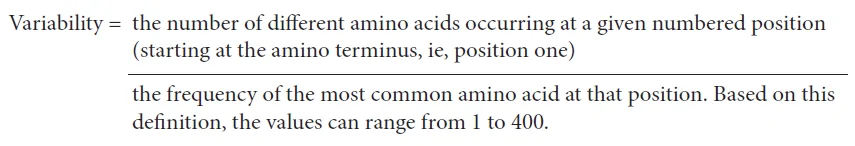



Using this definition, Wu and Kabat identified three stretches of relatively variable positions and 4 regions of lower sequence variability in the V domains of the kappa light chains they studied in this analysis. In later studies, the concept was extended to lambda light chain and heavy-chain V domains. Eventually, the 3 more variable stretches of amino acid sequence formally took on the labels “hypervariable regions” (HV) or “complementarity-determining regions” (CDR), and the 4 portions of the V domains with lower amino acid sequence variability were termed “framework regions” (FR).

It is important to note that the label HV is applied to positions based on data from the sequencing of the proteins, originally by Edman degradation of the actual polypeptide chains [12]. Thus, the assignment of “HV” to positions can only be based on the analysis of a population of immunoglobulins of different specificities.

In contrast and at least in principle, the label “complementarity-determining” can reasonably be used to refer to residues of a particular antibody in contact with a particular antigen in an individual complex. Alternatively, one could refer to complementarity-determining residues to denote amino acids of defined position in the primary structure, documented to be in contact with antigen in 1 or more solved crystal structures of different antibodies complexed with different antigens. Neither of these criteria has been used as such a basis for defining CDRs.

In practice, actual numbering and classification schemes for antibody variable domains rely on the sequence variability (as originally defined by Wu and Kabat) or on structural or topological criteria applied to the antibody V domain loops. So, despite the fact that “HV region” and “CDR” are often used interchangeably, their meanings in several of these schemes are generally overlapping but non-identical [13].

A further consideration is that “complementarity-determining” residues can reasonably be biophysically defined in more than one way. In other words, one can reasonably choose different criteria for what spatial and biophysical relationships between antibody and antigen atoms constitute “contact.”

One example is provided by Gorelik et al [14]. They note that identification of contact residues by the solvent-accessible surface area vs by interatomic distances of <4.0 Å can define non-identical surfaces and therefore non-identical lists of contact residues. Additional possible definitions for amino acid residues that help to determine complementarity could include non-contact amino acids that, when substituted with 1 or more alternative amino acids, structurally modify the antibody-antigen interface or alter the affinity [15].

As suggested above, in any given complex, the antibody amino acids that make van der Waals contact with antigen residues are only a subset of the amino acids in the 3 HV or CDR regions in the heavy- and light-chain V domains. Estimates for the percent of amino acids within HV or CDR regions that make van der Waals contact with antigen residues in any given antibody-antigen complex are generally between 20% and 33%, indicating that the majority of residues assigned to HV regions or CDRs are not serving as contact residues in any particular interface [16, 17]. Regarding the absolute number of residues assigned to CDRs, for 5 of the well-known CDR vs framework classification schemes, that magnitude varies somewhat, as illustrated in Table 1 [13].

**Table 1.** Different Schemes for Assigning CDRs.

Numbering Scheme	Key Criteria for Identifying CDRs	Number of Amino Acids					
		L1	L2	L3	H1	H2	H3
Kabat	Variability of aa at each position	11	7	9	5	16	8
Chothia	CDR loops based on analysis of Ab structures	11	7	9	10	5	8
Chothia Consensus	Standardization of different versions of Chothia analysis across more antibody structures	7	3	6	7	5	6
AbM	Similar to Chothia but with some corrections and refinements to best support homology modeling	11	7	9	10	9	8
IMGT	Universal aa numbering framework for conserved loop boundaries for Abs and TCRs	12	10	13	12	10	13

Abbreviations: aa, amino acid residue; Ab, antibody; CDR, complementarity-determining residues; TCRS, T cell receptors. This table is based on information in Table 2 and the text from Zhu et al [13].

Table 2 in the publication by Zhu et al [13], which presents the boundaries for the CDRs according to each of the 5 primary structure numbering schemes considered, indicates that the oldest system, Kabat, assigns a total of 55 residues to the 3 VL and 3 VH CDRs in the absence of insertions, which can occur in some VH or VL domains. In contrast, the widely used IMGT scheme, which also uses a distinct amino acid numbering method, assigns 70 residues to the 6 CDRs, again in the absence of insertions. For the technical details of some of the most widely used V domain numbering and CDR-defining schemes, the reader should consult the thorough review by Zhu et al and references cited therein.

## AMIT … POLJAK

Sixteen years after the publication of Wu and Kabat, Roberto Poljak and his colleagues published the first-ever crystal structure of an antibody Fab fragment complexed with a protein antigen, hen egg lysozyme (HEL) [18]. These authors studied a complex between the purified Fab fragments of the monoclonal antibody, D1.3, and the small globular protein HEL, the immunogen used to elicit D1.3-producing B cells in a mouse of an inbred strain. The resolution of the structure reported was 2.8 Å.

In the structural model based on the X-ray diffraction data, 17 amino acids of the antibody variable domains were identified as engaging in van der Waals contact with or hydrogen bonding to 16 amino acid residues of the lysozyme. Two contact residues from D1.3 were located outside the standard HV/CDR regions as defined by Kabat. These literal outlier residues illustrate how population-based and individual complex-based definitions of CDRs can differ.

For the D1.3 and HEL interface, the maximum dimensions are 30 by 20 Å. The total buried surface area on the antigen was 748 Å^2^ and on the antibody 690 Å^2^. Based on the end-on views of the respective antibody and antigen contact surfaces presented in Figure 3 of Amit et al, neither of these surfaces is completely contiguous.

This first structure for a paratope-protein epitope complex supports the expectation of Wu and Kabat that the antibody constructs the antigen-recognizing surface primarily from the VH and VL HV/CDR regions, which structurally correspond to loops that are found together at the distal ends of the Fab fragments in the tertiary structure. Many subsequent studies have offered additional support for this claim [reviewed as of 1994 in [16].

## KEY FEATURES OF D1.3-HEL COMPLEX

For this particular complex, contact residues of the antibody represented all 6 hypervariable regions in VH and VL, although only one residue from VL CDR2 makes a direct contribution to the van der Waals interface with lysozyme. In contrast, for many crystal structures of antibody-protein antigen complexes, not all HV/CDR regions contribute residues to the antibody-antigen contact interface [19].

Looking at individual residues in various of the canonical loop conformations defined originally by Chothia and colleagues [20, 21], it is only a small minority of amino acid residues that participate in contact more than 50% of the time. In MacCallum et al, of 11 residues contributing to CDR-H2 canonical class 1, 4 do not make contact with antigen in any of 6 complexes studied. Only 2 of the residues participate over 50% of the time. Four of the remaining 5 residues contribute to antigen contact less than 20% of the time. Similar results are seen for CDR-H2 canonical class 2 in studying 11 structures.

Based on the totality of information obtained about this complex, the Poljak team concluded that:

“The classical ‘lock-and-key’ metaphor is an adequate simplification to describe the interaction of lysozyme with antibody D1.3.”

Amit et al cite papers by Emil Fischer [22] and Paul Ehrlich [23] for the origin of the lock-and-key concept. Poljak and colleagues also mention observing only a single water molecule at the interface. Otherwise, the authors state that they did not make an effort to locate solvent molecules in or near the interface of the complex.

Although in this Historical Highlight article we will not delve into the full details of the next 2 original reports by Poljak and colleagues in 1990 [24] and 1994 [25], they reveal that the initial conclusions required significant revision. In these later studies, the authors determined the structures of crystals between HEL and D1.3 Fv as well as Fab fragments. An Fv contains only the VH and VL domains, leaving out the CH1 and CL domains included in an Fab fragment.

A difference, perhaps the key difference, between these further analyses and the crystal structure assessment of 1986 is that the atomic resolution was refined further. For the 1990 study, the resolutions were 2.5 Å for D1.3 Fab-HEL and 2.4 Å for D1.3 Fv-HEL [24]. In the 1994 analysis, the resolution improved to 1.8 Å for D1.3 Fv-HEL and free Fv [25].

Because of this improvement in the resolution of the analysis and in the associated ability to more definitively localize particular chemical features of small scale, the authors of the 1990 paper substantially altered the conclusion of the 1986 report that the D1.3-lysozyme complex illustrated the lock-and-key mode of interaction. The claim in the 1990 paper was that this complex was better understood as an example of induced fit, also sometimes referred to as adaptive fit. In other words, in the process of forming a complex, one or both molecular partners are subject to conformational adjustments. In this instance, the precise spatial relationship between VH and VL domains of the Fv D1.3 changes from the unbound Fv.

With the further improvement of the crystal structure resolution to 1.8 Å in 1994, the assessment of the extent of hydration at the interface between D1.3 FV and the antigen changed dramatically. In striking contrast to the relevant statements in the 1986 paper, the 1994 authors indicate that in addition to the retention at the interface of some bound waters from the unbound proteins, additional bound water molecules relative to those associated with the unbound species are recruited to the interface by the process of complex formation.

The 1994 authors state: “In all, 23 water molecules were located in the free antibody combining site and 48 at the antigen-antibody interface.” Also of significance, water molecules directly participate in hydrogen bonds with amino acids of the antigen and assist in creating the extent of the shape complementarity characterizing the paratope-epitope interface. Some of these inter-molecular bridges involve 2 or more water molecules.

Bound water molecules associated with proteins are thus, for some purposes, part of the functional unit, which is typically attributed solely to the “naked” protein whose amino acid sequence is directly encoded by a gene. In this perspective, the protein-bound water molecules are only indirectly encoded, a molecular subtext. The relationship between proteins, such as antibodies, and bound water molecules seems analogous to the relationship between a host animal, such as a human being, and that organism’s gut microbiome. As above, the gut microbiota are only indirectly encoded in an animal’s genome but still play a crucial role in the organism’s overall physiology.

A remarkably valuable aspect of the 1986 article by Poljak and colleagues is the information contained in 3 tables (Tables 1, 2, and 3). These tables identify the precise noncovalent contacts that define the D1.3-HEL interface and reveal that one amino acid from either the antibody or the antigen can have multiple atoms making contacts with the molecular partner. Furthermore, even a single atom can be involved in multiple van der Waals or other noncovalent interactions with atoms of the partner molecule. Amit et al note that most antigen residues that make contact with the antibody V domains interact with only 1 of the 2 V domains (ie, VH or VL, but not both), but 3 of the 16 antigen residues that contact the antibody engage with both VH and VL.

## SUMMARY

Of course, there are numerous additional studies by such eminent investigators as Michael Heidelberger, Karl Landsteiner, David Talmage, Henry Kunkel, and Alfred Nisonoff (and many others) that could have been used as the basis for a discussion of antibody-mediated recognition of antigen. The 3 articles we have focused on nevertheless provide a useful starting point for beginning to grasp the many intricacies of antibody binding and specificity.

As suggested by the quote from Linus Pauling at the start of this article, shape complementarity, as emphasized by Pauling and Delbrück in 1940, is an important feature of the antibody-antigen interaction surface, but there are other factors that influence the affinity of an antibody for a cognate ligand and the differential in affinity of an antibody for the cognate antigen and 1 or more non-cognate antigens. A subsequent article in the Historical Highlight series or elsewhere will be necessary to explore these other factors in depth and to achieve a deeper level of understanding of the biophysics of antibody recognition.

Wu and Kabat, in their study from 1980, introduced a quantitative measure of amino acid sequence variability and the concepts of hypervariable and framework residues in the VL domains of antibodies. These concepts have since been applied to VH domains of antibodies as well as T cell receptors and also, to some extent, other molecules.

The results of the first crystallographic study of an antibody Fab-protein antigen interaction in 1986 by Poljak and his colleagues yielded a number of insights that have been supported by the many subsequent studies of such complexes. Among the key findings that have been replicated are the following: 1) variable contributions to the area of contact with antigen by the VH vs VL; 2) unequal contributions of the individual HV regions to the buried surface area; 3) the potential participation of multiple atoms from one side chain; 4) the ability of one atom to make van der Waals contact with multiple atoms of the molecular partner; 5) the involvement of main chain atoms, not just side chain atoms, in intermolecular contact; 6) the substantial number of amino acid residues from both molecular species forming the noncovalent complex that participate in the interface of the complex; 7) the irregular shape of the interface; and 8) the potential for a non-contiguous antibody-antigen contact surface on either molecular participant.

There are, however, a number of important findings reiterated or added by later studies from multiple laboratories. These include: 1) the potential for significant conformational adjustments (alteration of either tertiary or quaternary structure) by either partner (antibody, antigen, or both), so-called adaptive fit; 2) the presence of significant numbers of water molecules in the bimolecular interface; 3) the ability of networks of water molecules to connect the two interacting proteins; 4) the potential for dozens of somatic mutations in the VH and VL to alter affinity and specificity for antigen; 5) the possibility of large insertions from non-immunoglobulin sequences into a V domain and that can contribute greatly to antigen binding affinity and specificity; 6) the simultaneous binding of a paratope to 2 different antigenic polypeptides on for example, a virion surface; 7) the simultaneous recognition by a paratope of both peptide and glycan components; 8) the possibility of noncovalent interactions between Fabs of distinct human IgG molecules that bind to epitopes in close proximity; and 9) the ability of a single antibody variable domain module (VH + VL) to bind, at different times, with meaningful affinities to 2 or more non-identical polypeptide antigens and/or non-polypeptide antigens, such as glycans or other chemical species.
