# Erratum to: Historical Highlight: Three Seminal Articles Relating to the Recognition of Protein Antigens by Antibodies

**DOI:** 10.20411/pai.v11i2.1136

**Published:** 2026-10-23

**Authors:** Neil S. Greenspan, Emily N. Kukan

**Affiliations:** 1 Case Western Reserve University

## Abstract

Erratum: In the article “Historical Highlight: Three Seminal Articles Relating to the Recognition of Protein Antigens by Antibodies” by Greenspan and Kukan, published in Pathogens and Immunity, Volume 11, Issue 2, pages 116-126 (doi: 10.20411/pai.v11i2.1091), the authors incorrectly interpreted the description of the method for calculating shape complementarity reported by Lawrence and Colman in “Shape Complementarity at Protein/Protein Interfaces.”

The original text stated that the measure of shape complementarity was “calculated for an arbitrary and randomly selected number of atoms at the interface.”

The text has been corrected to state that the measure of shape complementarity is “calculated for every antibody or antigen atom deemed to be in the interface.”

The authors regret the error and thank Mike Lawrence for providing his feedback. This correction does not otherwise affect the arguments in or conclusions of the article as originally articulated.

## ERRATUM

In the article “Historical Highlight: Three Seminal Articles Relating to the Recognition of Protein Antigens by Antibodies” by Greenspan and Kukan, published in *Pathogens and Immunity*, Volume 11, Issue 2, pages 116–126 (doi: 10.20411/pai.v11i2.1091), the authors incorrectly interpreted the description of the method for calculating shape complementarity reported by Lawrence and Colman in “Shape Complementarity at Protein/Protein Interfaces.”

The original text stated that the measure of shape complementarity was “calculated for an arbitrary and randomly selected number of atoms at the interface.” The text has been corrected to state that the measure of shape complementarity is “calculated for every antibody or antigen atom deemed to be in the interface.”

The authors regret the error and thank Mike Lawrence for providing his feedback. This correction does not otherwise affect the arguments in or conclusions of the article as originally articulated.

